# LncBook: a curated knowledgebase of human long non-coding RNAs

**DOI:** 10.1093/nar/gky960

**Published:** 2018-10-17

**Authors:** Lina Ma, Jiabao Cao, Lin Liu, Qiang Du, Zhao Li, Dong Zou, Vladimir B Bajic, Zhang Zhang

**Affiliations:** 1 BIG Data Center, Beijing Institute of Genomics, Chinese Academy of Sciences, Beijing 100101, China; 2 CAS Key Laboratory of Genome Sciences and Information, Beijing Institute of Genomics, Chinese Academy of Sciences, Beijing 100101, China; 3 University of Chinese Academy of Sciences, Beijing 100049, China; 4 Anhui University of Technology, Maanshan 243032, China; 5 King Abdullah University of Science and Technology (KAUST), Computational Bioscience Research Center (CBRC), Computer, Electrical and Mathematical Sciences and Engineering Division (CEMSE), Thuwal 23955-6900, Kingdom of Saudi Arabia

## Abstract

Long non-coding RNAs (lncRNAs) have significant functions in a wide range of important biological processes. Although the number of known human lncRNAs has dramatically increased, they are poorly annotated, posing great challenges for better understanding their functional significance and elucidating their complex functioning molecular mechanisms. Here, we present LncBook (http://bigd.big.ac.cn/lncbook), a curated knowledgebase of human lncRNAs that features a comprehensive collection of human lncRNAs and systematic curation of lncRNAs by multi-omics data integration, functional annotation and disease association. In the present version, LncBook houses a large number of 270 044 lncRNAs and includes 1867 featured lncRNAs with 3762 lncRNA–function associations. It also integrates an abundance of multi-omics data from expression, methylation, genome variation and lncRNA–miRNA interaction. Also, LncBook incorporates 3772 experimentally validated lncRNA-disease associations and further identifies a total of 97 998 lncRNAs that are putatively disease-associated. Collectively, LncBook is dedicated to the integration and curation of human lncRNAs as well as their associated data and thus bears great promise to serve as a valuable knowledgebase for worldwide research communities.

## INTRODUCTION

Long non-coding RNAs (lncRNA) have a variety of functions in many important biological processes (1–5) and are closely associated with various diseases (6–9). In recent years, rapid advances of next-generation sequencing technologies have triggered an explosion of newly discovered lncRNAs (especially in human) (9–12), primarily due to their highly tissue/cell-specific (5,13–15) and lineage/species-specific (14,16,17) nature. Accordingly, multiple databases have been constructed to archive lncRNA sequences and annotations (10,12,14,18,19), collect experimentally validated lncRNAs (2,3,9,20), curate lncRNAs-disease associations (6,7,9), and annotate miRNA–lncRNA interactions (21–23). Despite this, lncRNAs are still poorly annotated (24), posing great challenges for better understanding their functional significance and dissecting their complex functioning molecular mechanisms.

To harness collective intelligence for gathering and annotating human lncRNAs, we constructed LncRNAWiki (9) in 2015, a wiki-based platform for community curation of human lncRNAs. LncRNAWiki has frequently been updated by adding more experimentally validated lncRNAs, incorporating small peptides encoded in lncRNAs and associating lncRNAs with diseases (25,26). However, LncRNAWiki, built based on MediaWiki, has significant limitations on managing structured data and providing customized functionalities; functional annotations and sequence data are stored as unstructured text in MediaWiki, which makes it difficult to retrieve and show data items of interest. It would be desirable to organize large-scale annotations in a structured manner and to provide customized web functionalities with more friendly interfaces. More importantly, it is highly beneficial to integrate multi-omics data with the aim to significantly enrich and improve lncRNAs’ annotations to support function inference.

Here we develop an expert-curation-based resource, LncBook (http://bigd.big.ac.cn/lncbook), as a complement to community-curation-based LncRNAWiki. LncBook features a comprehensive collection of human lncRNAs and systematic curation of lncRNAs by multi-omics data integration, functional annotation and disease association (Table 1). It houses a larger number of human lncRNAs that are not only derived from existing databases but also novel RNA assemblies based on RNA-seq data analysis. It includes community-contributed annotations from LncRNAWiki and expert-curated annotations curated from published literature, respectively. Particularly, it integrates a variety of multi-omics data, including expression, methylation, variation, and interaction, conducts functional annotation and incorporates a collection of lncRNA-disease associations. Equally important, LncBook organizes all relevant data in a structured manner, facilitating data browse/search with more enhanced efficiency and provides several useful tools for online analysis.

**Table 1.** Key differences between LncRNAWiki 2015 and LncBook

Data Item	LncRNAWiki 2015	LncBook
LncRNA transcripts	105 255	**270 044**
Featured lncRNAs	86	**1867**
LncRNA–function associations	NA	**3762**
LncRNA-disease associations	NA	**3772**
Predicted disease-associated lncRNAs	NA	**97 998**
HK and TS lncRNAs	NA	**819 HK**
		**49 115 TS**
Methylation profiles	NA	**Profiles in nine cancers**
Genome variations	NA	**92 725 757 SNPs**
LncRNA-miRNA interactions	NA	**129 690 817 predicted lncRNA–miRNA interactions**
Tools	BLAST	**BLAST, classification, coding potential prediction, ID conversion**

## MATERIALS AND METHODS

### Data collection

**Figure 1.**
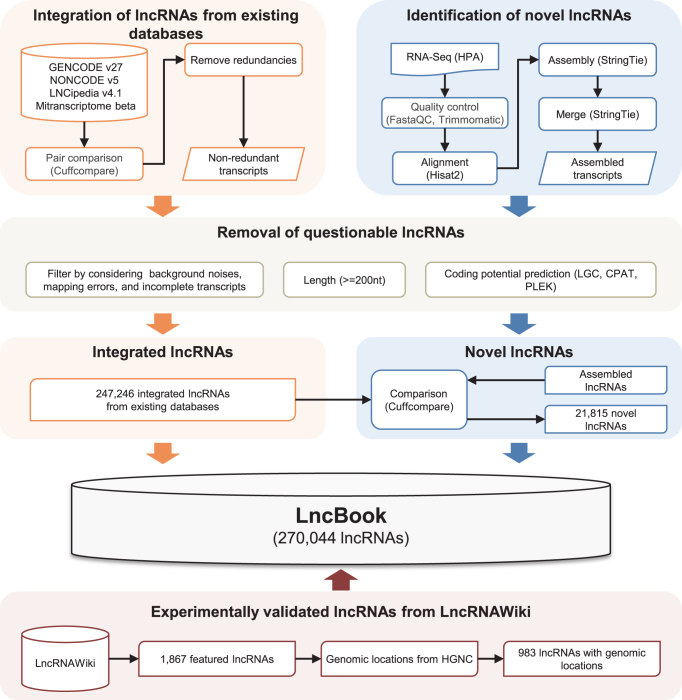
Workflow diagram of lncRNA curation and integration. The human lncRNAs in LncBook are compiled from (i) existing databases, (ii) novel RNA assemblies based on RNA-seq data analysis and (iii) published literature.

The human lncRNAs in LncBook were obtained not only from existing databases and published literature but also from novel RNA assemblies based on RNA-seq data analysis (Figure 1). Namely, we collected lncRNAs from several well-known lncRNA databases, including GENCODE v27 (14), NONCODE v5.0 (12), LNCipedia v4.1 (10) and MiTranscriptome beta (11). To obtain high-confidence lncRNAs, a set of strict criteria was adopted considering redundancy, background noise, mapping error, incomplete transcripts, length and coding potential. We used Cuffcompare (27) to compare different datasets to remove redundant, questionable or incomplete transcripts: (i) redundant transcripts were identified using Cuffcompare (27) with the comparison code ‘ = ’ (which means complete match of intron chain), and then representative lncRNAs were selected according to their annotation quality; (ii) questionable transcripts were detected using Cuffcompare (27) with the comparison codes ‘e’, ‘p’ and ‘s’. Single-exon transcripts that are part of multi-exon transcripts and located in their exon regions, were regarded as incomplete lncRNAs. Also, transcripts with very short exons (<15 nt) at the 5′ and 3′ ends were considered as incomplete lncRNAs. In addition, transcripts shorter than 200 nt were excluded. Moreover, three algorithms, namely, LGC (an in-house tool publicly available at http://bigd.big.ac.cn/biocode/tools/BT000004), CPAT (28) and PLEK (29), were used for coding potential estimation, and we only retained transcripts that were identified as lncRNAs by all the three algorithms. As a consequence, an integrated, non-redundant and high-quality dataset containing 247 246 lncRNAs was obtained.

To identify novel lncRNAs, we downloaded 122 RNA-seq datasets from HPA (Human Protein Atlas) (30). FastaQC (https://www.bioinformatics.babraham.ac.uk/projects/fastqc/) and Trimmomatic (31) were used for quality control. Hisat2 (32) was used for reads mapping, while StringTie (32) was used for assembling and merging transcripts. We compared the assembled transcripts with existing lncRNAs using Cuffcompare (27). As a result, a total of 21 815 novel lncRNAs were identified. At last, we integrated a large collection of lncRNAs from existing databases, novel RNA assemblies based on RNA-seq data analysis, and literature reported lncRNAs that were obtained from LncRNAWiki (9). After removing lncRNAs that were not traceable back to their genomic locations, we finally obtained a total of 270 044 non-redundant lncRNA transcripts, belonging to 140 362 gene loci. All these lncRNAs are publicly available at ftp://download.big.ac.cn/lncbook.

### Data integration and annotation

To profile expression levels for lncRNAs, two RNA-seq datasets were used: HPA (Human Protein Atlas, 32 normal human tissues are covered) (30) and GTEx (Genotype-Tissue Expression, 53 normal human tissues are covered) (33). We filtered out lncRNAs whose highest expression values are lower than 0.5 TPM/FPKM. Then, τ-value (34) and cv (coefficient of variance) value were used to determine HK (housekeeping) lncRNAs (τ-value ≤ 0.5 and cv ≤ 0.5) and TS (tissue-specific) lncRNAs (*τ*-value ≥ 0.95). To annotate methylation information of lncRNAs, bisulfite-seq data from TCGA (The Cancer Genome Atlas) (https://portal.gdc.cancer.gov) and ENCODE (The ENCyclopedia of DNA Elements) (https://www.encodeproject.org) were downloaded, covering nine cancers with both normal and cancer samples. We defined regions from −1500 bp relative to the transcription start sites as promoters, and calculated the methylation levels of both promoter and body regions of lncRNAs. In addition, we mapped the SNP sites in dbSNP (35) to the lncRNA loci, and annotated MAF (Minor Allele Frequency) values based on 1000 Genomes Project (36), pathogenic information in ClinVar (version 2017.9.05) (37) and COSMIC (version 85) (38) using ANNOVAR (39). TargetScan (40) and miRanda (41) were used to predict lncRNA–miRNA interactions and experimentally validated interactions were obtained from starBase v2.0 (21).

To provide high-quality annotations for experimentally validated lncRNAs, we systematically curated 1867 lncRNAs (that were sourced from LncRNAWiki (9)) with function annotations reported in 2501 publications and with controlled vocabularies describing their functioning mechanisms and biological processes they affect.

The associations between lncRNA and disease were derived from LncRNADisease (6) and LncRNAWiki (9), which have been extracted from published literature with experimental evidence. Each lncRNA-disease association was annotated with disease name, MeSH Ontology (Medical Subject Headings 2018 name), dysfunction type, detailed description and publication. On the other hand, we predicted disease-associated lncRNAs according to the evidence from methylation, genome variation and lncRNA–miRNA interaction: (i) Methylation: In each cancer, lncRNA whose promoter region methylation level shows increase (decrease) in 80% cancer samples relative to normal samples is considered to be hypermethylated (hypomethylated). Thus, we considered one lncRNA to be cancer-associated if it is consistently hypermethylated or hypomethylated in at least eight cancers; (ii) Genome Variation: any lncRNA overlapping disease-associated SNPs of COSMIC (OCCURRENCE ≥ 3) or ClinVar in its genomic location we considered to be disease-associated; (iii) Interaction: any lncRNA interacting with at least 11 disease-associated miRNAs (associated with at least five diseases according to the Human microRNA Disease Database HMDD (42)) we considered to be disease-associated.

### Implementation

We developed LncBook using String Boot as back-end web framework and MySQL (http://www.mysql.org) as database engine. Web interfaces were developed by JSP (Java Server Pages) and AJAX (Asynchronous JavaScript and XML). Bootstrap (https://getbootstrap.com) was adopted as a front-end framework, which provides a series of templates for designing web pages with consistent interface components. Also, data visualization was powered by Highcharts (a charting library written in pure JavaScript), offering an easy way of adding interactive charts to any web site or application.

## DATABASE CONTENTS AND FEATURES

Compared with the existing lncRNA databases, LncBook features a comprehensive collection of human lncRNAs and systematic curation of lncRNAs’ annotation by multi-omics data integration, function annotation and disease association (Table 1). In the current version, LncBook houses a total of 270,044 lncRNA transcripts, contains 1867 experimentally validated lncRNAs manually curated based on published literature, and annotates all lncRNAs by integration of large-scale multi-omics data including tissue expression profiles, cancer-associated methylation levels, genome variations and lncRNA–miRNA interactions. These 1867 featured lncRNAs that have been documented in published literature are systematically curated and annotated with functioning mechanisms and biological processes, resulting in 3762 lncRNA–function associations. LncBook also includes a total of 3772 experimentally validated lncRNA-disease associations and identifies 97 998 lncRNAs that are putatively associated with diseases. Also, a series of useful tools, such as coding potential prediction, sequence search, etc., are deployed in LncBook.

### Comprehensive collection of human lncRNAs

**Figure 2.**
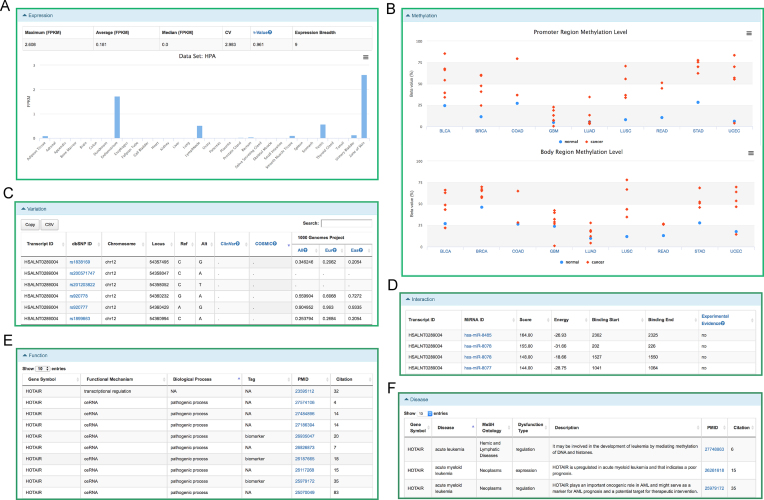
Screenshots of web pages for a lncRNA transcript. For example, *HOTAIR* is extensively annotated with an abundance of multi-omics data including (**A**) expression, (**B**) methylation, (**C**) variation, (**D**) interaction and the systematic curations of (**E**) function and (**F**) disease based on published literature.

LncBook accommodates a comprehensive collection of 270 044 human lncRNAs (see details in ‘Materials and Methods’ section), including 247 246 lncRNAs obtained from existing databases, 1867 from LncRNAWiki and 21 815 novel lncRNAs identified based on RNA-seq data analysis, which together belong to 140 362 gene loci. LncBook manages human lncRNAs based on transcripts, where a unique accession number prefixed with HSALNT is assigned to each lncRNA transcript entity. Likewise, the lncRNA gene has an accession number prefixed with HSALNG. In LncBook, each transcript corresponds to a specific web page containing basic information (symbol, genomic context, length, exon number, GC content, classification, sequence, longest ORF length, coding potential), multi-omics data (expression, methylation, genome variation, lncRNA–miRNA interaction), function annotations and disease associations (Figure 2).

### Multi-omics data integration

LncBook integrates a variety of multi-omics data, enriching lncRNAs with abundant annotations in expression, methylation, genome variation and interaction with miRNAs (see details in ‘Materials and Methods’ section). For any given lncRNA, LncBook profiles its expression levels across all collected tissues and visualizes its expression profile in a bar chart, greatly facilitating users to explore functional significance. Based on these expression profiles across different tissues, LncBook further identifies a total of 819 HK lncRNAs, which are consistently expressed in almost all tissues. Similarly, it also obtains 49 115 TS lncRNAs, which are expressed specifically in one or few tissues. All HK and TS lncRNAs are publicly available at http://bigd.big.ac.cn/lncbook/expression. Also, for each lncRNA, LncBook provides methylation levels of both promoter and body regions in normal and cancer samples across nine cancers, which are summarized in a table and visualized in a dot plot. According to the methylation analysis results, only 583 lncRNAs are always hypermethylated in cancers, contrasting to 27 723 lncRNAs that are always hypomethylated. LncBook also collects 92 725 757 SNPs from dbSNP (35) residing in 197 799 lncRNA transcripts. Among all these SNPs, there are 7571 pathogenic SNPs from ClinVar (37) overlapping 2280 lncRNA transcripts and 79 012 pathogenic SNPs from COSMIC (38) (OCCURRENCE ≥ 3) overlapping 26 008 lncRNA transcripts. Also, LncBook includes 145 lncRNA–miRNA interactions supported by experimental evidence from starBase (21), as well as 129 690 817 interactions predicted by TargetScan (40) and miRanda (41).

### Function annotation

Although a large number of lncRNAs have been identified in human, only a small fraction of them have experimental evidence with supported publications. According to the current collection of LncBook, there are only 1867 out of all 270 044 lncRNAs that have been documented experimental validation. Based on manual curation of 2632 publications, LncBook provides comprehensive function annotations for these 1867 featured lncRNAs; 1653 lncRNAs have function annotation, while 1502 lncRNAs are linked to different diseases, leading to 3762 lncRNA–function associations. Specifically, each lncRNA–function association in LncBook is described using controlled vocabularies in light of functioning mechanism and biological process in which they are involved. Regarding functioning mechanism, LncBook adopts six controlled terms with each having different number of associations: transcriptional regulation (397 associations), ceRNA (182 associations), splicing regulation (19 associations), translational control (17 associations), protein localization (4 associations) and RNAi (3 associations). For biological process, LncBook adopts two terms, namely, pathogenic process and developmental process; function annotation of featured lncRNAs shows that most of them are involved in cancer and other diseases (3598 associations), compared to developmental process (53 associations).

### LncRNA-disease association

Considering that most of the functionally studied lncRNAs are closely associated with human diseases, LncBook integrates 3772 lncRNA-disease associations, derived not only from LncRNADisease (6) and LncRNAWiki (9) but also curated based on 2337 publications. LncBook describes each association with disease name, dysfunction type, detailed description, MeSH disease ontology and publication. According to the current information contained in LncBook, all lncRNAs in LncBook are associated with 462 diseases and 28 MeSH disease terms. Among all the terms, ‘Neoplasms’ has the largest number of associations (2888 associations), followed by the term ‘Digestive System Diseases’ that has 888 associations. LncBook-contained information also reveals that among all the disease-associated lncRNAs, *HOTAIR, MALAT1, H19, MEG3, CDKN2B-AS1, PVT1, NEAT1* and *GAS5* are extensively studied and each of them is associated with at least 30 different diseases.

Additionally, based on an abundance of methylation, genome variation and lncRNA–miRNA interaction, LncBook predicts a total of 97 998 lncRNAs that are potentially associated with diseases (see details in ‘Materials and Methods’ section). Briefly speaking, one lncRNA is putatively believed to be disease-associated only if any evidence for that can be obtained from methylation, genome variation and/or lncRNA–miRNA interaction. For a specific lncRNA under investigation, supporting evidence can be that, for example, its methylation change relates to disease, it overlaps pathogenic variations, or it frequently interacts with disease-associated miRNAs. As a consequence, LncBook contains a collection of 97 998 disease-associated lncRNAs, where 607 are supported by three sources of evidence, namely, methylation, genome variation and lncRNA–miRNA interaction, 13 257 are supported by two of them, and 84 134 are supported by only one of them. All these disease-associated lncRNAs can be found at http://bigd.big.ac.cn/lncbook/disease.

## DISCUSSION AND FUTURE DIRECTIONS

LncBook is dedicated to the integration and curation of human lncRNAs as well as their associated data. In harmony with LncRNAWiki that is a community-curated resource, LncBook serves as an expert-curated knowledgebase that integrates a comprehensive collection of human lncRNAs and contains multi-omics data, function annotations and disease associations. The current implementation of LncBook houses a large number of 270 044 lncRNAs and includes 1867 featured lncRNAs with 3762 lncRNA–function associations. It also integrates an abundance of multi-omics data from expression, methylation, genome variation and lncRNA–miRNA interaction. Also, LncBook includes 3772 experimentally validated lncRNA-disease associations and identifies 97 998 lncRNAs that are putatively disease-associated. However, of note, this does not mean that these disease-associated lncRNAs play causative roles in diseases (24). Taken together, LncBook is a curated knowledgebase of human lncRNAs and has the potential to serve as a valuable resource for worldwide research communities. Future developments of LncBook include regular integration of newly discovered lncRNAs, incorporation of high-quality annotations through literature curation and identification of differentially expressed lncRNAs in normal and disease samples. We also plan to integrate full-length lncRNAs from additional databases such as FANTOM CAT (43) and BIGTranscriptome (44). In addition, more user-friendly tools will be developed in aid of functional annotation and interactive visualization of various omics data. We also look forward to comments and suggestions from researchers worldwide, aiming to build LncBook into an encyclopedia of human lncRNAs.
