# LncBook: a curated knowledgebase of human long non-coding RNAs

**DOI:** 10.1093/nar/gkz073

**Published:** 2019-01-31

**Authors:** Lina Ma, Jiabao Cao, Lin Liu, Qiang Du, Zhao Li, Dong Zou, Vladimir B Bajic, Zhang Zhang

**Affiliations:** 1 BIG Data Center, Beijing Institute of Genomics, Chinese Academy of Sciences, Beijing 100101, China; 2 CAS Key Laboratory of Genome Sciences and Information, Beijing Institute of Genomics, Chinese Academy of Sciences, Beijing 100101, China; 3 University of Chinese Academy of Sciences, Beijing 100049, China; 4 Anhui University of Technology, Maanshan 243032, China; 5 King Abdullah University of Science and Technology (KAUST), Computational Bioscience Research Center (CBRC), Computer, Electrical and Mathematical Sciences and Engineering Division (CEMSE), Thuwal 23955–6900, Kingdom of Saudi Arabia

*Nucleic Acids Research*, 2019, Vol. 47, Database issue, Pages D128–D134, https://doi.org/10.1093/nar/gky960

On page D131 the sentence: ‘According to the methylation analysis results, only 583 lncRNAs are always hypomethylated in cancers, contrasting to 27 723 lncRNAs that are always hypermethylated’ has been corrected to read ‘According to the methylation analysis results, only 583 lncRNAs are always hypermethylated in cancers, contrasting to 27 723 lncRNAs that are always hypomethylated.’

